# Anti-SARS-CoV-2 antibody among SARS-CoV-2 vaccinated vs post-infected blood donors in a tertiary hospital, Bangkok, Thailand

**DOI:** 10.1371/journal.pone.0285737

**Published:** 2023-05-18

**Authors:** Parichart Permpikul, Surat Tongyoo, Chutikarn Chaimayo, Prapan Kanpai, Jitmanee Virat, Sutasinee Virat, Jaratsri Chuchaaim, Anchalee Thongput, Sonu Bhatnagar

**Affiliations:** 1 Department of Transfusion Medicine, Faculty of Medicine Siriraj Hospital, Mahidol University, Bangkok, Thailand; 2 Department of Medicine, Faculty of Medicine Siriraj Hospital, Mahidol University, Bangkok, Thailand; 3 Department of Microbiology, Faculty of Medicine, Siriraj Hospital Mahidol University, Bangkok, Thailand; 4 Scientific Affairs, Abbott Laboratories Singapore Pte Ltd., Singapore, Singapore; University of Ilorin, NIGERIA

## Abstract

SARS-CoV-2 virus infection has imposed a significant healthcare burden globally. To contain its spread and decrease infection-related mortality, several vaccines have been deployed worldwide in the past 3 years. We conducted a cross-sectional seroprevalence study to assess the immune response against the virus among blood donors at a tertiary care hospital, Bangkok, Thailand. From December 2021 to March 2022, total of 1,520 participants were enrolled, and their past history of SARS-CoV-2 infection and vaccination was recorded. Two serology test, namely, quantitative IgG spike protein (IgG_SP_) and qualitative IgG nucleocapsid antibody (IgG_NC_) were performed. The median age of study participants was 40 years (IQR 30–48) and 833 (54.8%) were men. Vaccine uptake was reported in 1,500 donors (98.7%) and 84 (5.5%) reported the past infection history. IgG_NC_ was detected in 46/84 donors with the past infection history (54.8%) and in 36 out of the rest 1,436 (2.5%) with no past history. IgG_SP_ positivity was observed in 1484 donors (97.6%). When compared to unvaccinated donors (n = 20), IgG_SP_ level was higher in the donors who had received one vaccine dose (p< 0.001) and these antibody levels increased significantly among those with 3^rd^ and 4^th^ vaccine doses. Factors associated with low IgG_SP_ (lowest quartile) by multivariate analysis included: no past infection history, homologous vaccination, < 3 vaccine doses, and > 90 days duration since last vaccination. In conclusion, vaccine uptake among our study donors was high (98.7%) and IgG_SP_ antibody was observed in nearly all the vaccinated donors (97.6%). Previous SARS-CoV-2 infection, use of heterologous vaccination, vaccines ≥ 3 doses, and duration of the last vaccination >90 days affected IgG_SP_ levels. Use of serological assays were found beneficial in the evaluation and differentiation of immune response to vaccination, and natural infection including the identification of previous asymptomatic infections.

## Introduction

The SARS-CoV-2 virus has infected hundreds of millions of people globally and caused major fatalities (in millions) around the world [1]. Thailand reported its first case in early 2020 and since then, more than 4 million cases and nearly 30,000 deaths have been reported in the country [2]. To contain the SARS-CoV-2 pandemic, several vaccines were rapidly developed and implemented worldwide [3, 4]. The availability of various types of vaccines varied greatly among the countries and so does their timing [5]. To counter that, many countries devised novel and innovative ways to protect the more vulnerable groups and maximize the impact of available vaccines. Adoption of the heterologous booster vaccine practice is one such example [6, 7]. Beside that, various SARS-CoV-2 antibody assays were also developed which helped to evaluate immune response either from natural infection or vaccination [8, 9]. The antibody assays designed to detect antibodies against receptor binding domain (RBD) protein or spike protein (SP) correlated well with neutralizing antibody titres (or protective antibody) produced by both i.e. natural infection or vaccination [10]. On the other hand, the assays designed to detect antibodies against nucleocapsid protein (NC) could be used to identify natural infection specifically and hence measure the seroprevalence among the target population. Both anti-SP and anti-NC seroreactivity was affected by clinical severity of infection and duration between blood sampling and infection/vaccination date [11]. The waning of antibodies over time was reported for both anti-SP and anti-NC antibodies as will be expected in any respiratory infections [12]. There are numerous SARS-CoV-2 serological studies which reported that the use of heterologous booster vaccines was superior to the homologous booster vaccine in terms of immune response generation but the type of heterologous vaccines used varied among the different studies [6, 7, 13]. Seroprevalence data from Thailand have been limited and mainly focused on specific populations such as healthcare personnel [14–16]. Most of these studies were conducted during early 2020–2021, before the wide vaccine coverage and hence reported low seropositive prevalence in the country [14–16]. In the middle of 2021, covid vaccines became widely available in Thailand and there was also increase in infection cases identified after several waves of infection in the country [2]. Generally, blood donation samples are suitable for sero-surveillance because numerous samples can be efficiently collected and tested [17]. Therefore, this study was carried out to measure the seroprevalence of SARS-CoV-2 antibodies among blood donors and to characterize the antibody response to SARS-CoV-2 infection and vaccination in 2021–22 timeframe. We also explored the effects of potential correlates of immune activity, including age, time since the last vaccination received, various vaccine regimens, and the number of vaccine doses received on the nature of this response. The relationships between these variables and the relative quantification of antibodies against SARS-CoV-2 were also examined.

## Materials and methods

This cross-sectional study was approved by the Institutional Review Board of Siriraj Hospital, Faculty of Medicine, Mahidol University, Bangkok, Thailand (IRB no. *Si 653/2021*). All whole blood and platelet donors aged 18 years and above who qualified under the standard blood donor selection criteria and provided the written consent for study enrollment were included. Information was obtained regarding the donor demographics, history of past SARS-CoV-2 infection, and detailed history of COVID-19 vaccination. Serological data from study specimens were studied using quantitative and qualitative SARS-CoV-2 IgG_SP_ and IgG_NC_ assays respectively.

### IgG_SP_ assay

Quantitative SARS-CoV-2 IgG II tests were performed on the Abbott Alinity i platform (Abbott Laboratories, IL) following the manufacturer’s instructions. In this CMIA antibody test, SARS-CoV-2 antigen coated paramagnetic microparticles bind to IgG antibodies that attach to the virus spike protein in human serum and plasma samples. The resulting chemiluminescence in relative light units (RLU) following the addition of anti-human IgG acridinium labeled conjugate compared to the IgG II calibrator/standard indicates the strength of the response, which reflects the quantity of IgG_SP_ present. 50 AU/mL and above in this test are considered positive. This quantitative measurement of IgG_SP_ can be helpful to evaluate an individual’s humoral response to vaccines. The assay reported a LOD equal to 6.8 AU/ml. The First WHO International Standard NIBSC Code 20–136 showed the mathematical relationship of the Abbott AU / ml unit to the WHO BAU / ml unit as BAU/mL = 0.142 x AU/mL.

### IgG_NC_ assay

The Abbott Alinity i SARS-CoV-2 antinucleocapsid protein IgG assay (Abbott Laboratories, IL) is a semiquantitative CMIA assay for qualitative detection of IgG in human serum or plasma against the SARS-CoV-2 nucleoprotein. The qualitative results and the index values reported by the instrument were used in the analysis and the index values (S/co) of 1.4 and above are considered positive according to the manufacturer’s instructions. IgG_NC_ can be helpful to differentiate the humoral response from natural infection versus post vaccination.

### Donor deferral criteria with respect to SARS-CoV 2 infection and vaccination

During the study period, we deferred the prospective donors for one week after covid-19 vaccination and two weeks if there was any side effects reported after vaccination. After covid-19 infection diagnosed by positive SAR-CoV-2 from the nasopharyngeal swab, donors were deferred for 28 days from the day of diagnosis.

### Definition of SARS-CoV-2 Infections

From the beginning of the COVID-19 pandemic throughout the study period, all SARS-CoV-2 infection in Thailand was defined by the detection of the SARS-CoV-2 virus by molecular assay using nasopharyngeal swab sample [18].

### Statistical methods

The results of antibody tests and donor data from the case study form were used to identify the number of donors who had positive antibody tests and whether they had previously reported infection or contact history and vaccination. Data analysis and graphics were performed using Microsoft Excel and SPSS Statistics version 18 (SPSS.Inc., Chicago, IL). Continuous data are presented as median with interquartile range (IQR) and categorical data are expressed as percentages. We subdivided the population of IgG_SP_ positive donors into high and low antibody levels based on their IgG_SP_ level. Donors were assigned to the low antibody level group if their IgG_SP_ level was within the lowest IgG_SP_ quartile, while those who had an IgG_SP_ level within the upper 3 quartiles were classified as the high antibody level group.

To determine the predictive factor associated with lower IgG_SP_ levels, a comparative analysis was then performed between the low and high IgG_SP_ groups. For the comparison of non-normally distributed continuous variables, the Mann-Whitney U test was used. The chi-square test or Fischer’s exact test was used to compare categorical variable. A p-value less than 0.05 (<0.05) was considered statistically significant. Univariate analysis was performed to identify possible predictors associated with low IgG_SP_. Variables with a p-value less than 0.05 in univariate analysis were included in multivariate analysis. Multivariate binary logistic regression was used to identify predictors that were associated with low IgG_SP_ levels.

## Results

From December 2021 to March 2022, 1520 donors were enrolled. The median age was 40 years (IQR 30–48) and 833 donors (54.8%) were male. As noted in Table 1, most of them lived in Bangkok (58.7%), while others came from Bangkok suburbs, namely Nonthaburi (17%), Nakorn Pathom (5.3%), Samut Prakarn (3.5%), Pathum Thani (2.4%), Samut Sakorn (1.9%), Supanburi (1.2%) Chonburi (1.1%), and other provinces (8.9%). A total of 1500 donors (98.7%) were vaccinated and 84 (5.5%) reported previous COVID-19 infection. Of those who were vaccinated, 1480 (98.7%) had detectable IgG_SP_ antibody levels. Among 20 nonvaccinated donors, none of them reported a history of infection, and their both IgG_SP_ and IgG_NC_ antibody levels were low (Table 2).

**Table 1:** Donor baseline demographic data.

Donors’ characters	Overall N = 1520
Age, years, median (IQR)	40 (30–48)
Sex, male, n (%)	833 (54.8)
Previous SARS-CoV-2 infection, n (%)	84 (5.5)
Resident area, n (%)	
Bangkok	892 (58.7)
Nonthaburi	258 (17.0)
Nakorn Pathom	81 (5.3)
Pathum Thani	37 (2.4)
Samut Prakarn	53 (3.5)
Samut Sakorn	29 (1.9)
Supanburi	18 (1.2)
Chonburi	17 (1.1)
Others	135 (8.9)

**Table 2:** IgG_NC_ and IgG_SP_ level.

Clinical parameters	N	IgG_NC_ S/co index Median [IQR]	*p- value	IgG_SP_ AU/ml Median [IQR]	*p- value
All donors	1,520	0.08 [0.04–0.23]		4,393.6 [1,042.6–15,971.9]	
Age group (yrs)					
≤ 30	388	0.11 [0.05–0.33]		4,383.9 [1,222.6–17,584.6]	
31–50	862	0.08 [0.04–0.21]	0.001	4,679.4 [1,069.5–16,197.6]	0.372
>50	270	0.06 [0.04–0.13]	0.001	3,213.4 [837.0–13,503.9]	0.067
Sex					
Male	833	0.08 [0.04–0.20]		4,088.1 [913.4–14,560.7]	
Female	687	0.09 [0.04–0.25]	0.149	4,693.7 [1,053.4–17,594.1]	0.732
SARS-CoV-2 infection					
Past infection	84	1.57 [0.64–2.67]		7,051.6 [2,733.1–15,189.6]	
No infection	1,436	0.08 [0.04–0.19]	<0.001	4,185.3 [998.4–16,008.5]	0.551
Vaccination					
No vaccine	20	0.05 [0.02–0.09]		0.8 [0.00–7.0]	
1 dose	20	0.06 [0.03–0.30]	0.738	2,748.9 [926.1–10,159.7]	<0.001
2 doses	634	0.06 [0.03–0.20]	0.139	979.2 [437.6–2,250.2]	0.020
3 doses	698	0.09 [0.05–0.23]	<0.001	9,817.7 [4,223.1–20,352.6]	<0.001
4 doses	145	0.13 [0.06–0.29]	0.006	25,160.8 [16,722.1–40,000]	<0.001
5 doses	3	0.29 [0.21–0.49]	0.415	10,006.7 [53,24.2–23,525.9]	0.186
Duration since last vaccine					
≤90 days	894	0.08 [0.04–0.23]		11,154.7 [3,100.1–23,371.8]	
>90 days	594	0.09 [0.04–0.23]	0.877	1,377.8 [521.7.1–4,195.3]	<0.001
Vaccine regimens					
Homologous	510	0.06 [0.03–0.18]		849.0 [379.6–1,962.0]	
Heterologous	888	0.10 [0.05–0.24]	<0.001	9,284.4 [3,428.4–28,646.00]	<0.001
Vaccine type (2^nd^ & 3^rd^ doses)					
Whole inactivated virus (SP/SV)	539	0.13[0.07–0.30]		8,156.7[3,658.6–20,106.7]	
AZ/PZ/MD	237	0.06[0.03–0.11]	<0.001	16,435.8[9,824.0–27,977.2]	<0.001

*The p-value is the comparison between the row above in the same column. Homologous—same vaccine used for all vaccine dose; Heterlogous -different vaccine used for any subsequent vaccine dose. SP-Sinopharm;SV-Sinovac;AZ-AstraZeneca;PZ-Pifzer;MD-Moderna

The levels of IgG_NC_ and IgG_SP_ antibodies of the study population are stated in Tables 2 and 3. Overall, high positivity (1484/1520; 97.6%) was observed for IgG_SP_ with a median serological value of 4393.6AU/ml (IQR 1,042.6–15,971.9) among the study population. No statistically significant differences were observed in IgG_SP_ levels between different age groups and gender. For IgG_NC_, donors aged ≤ 30 years had higher S/co index values compared to those aged 31–50 years (0.11 [0.05–0.33] vs 0.08 [0.04–0.21], p = 0.001) and the latter group had higher S/co index values compared to donor aged >50 years (0.08 [0.04–0.21] vs 0.06 [0.04–0.13], p = 0.001). There was no statistically significant difference observed for IgG_NC_ antibodies among males and females. The value of the IgG_NC_ S/co index was higher in donors with a history of COVID-19 infection (1.57 [0.64–2.67] vs 0.08 [0.04–0.19]; p<0.001). However, no statistically significant difference was observed in their IgG_SP_ levels (p = 0.551). The effect of COVID-19 vaccination is also stated in Table 2. The level of IgG_SP_ was higher in donors who had received one dose of vaccination compared to unvaccinated (0.8 [0.00–7.0] vs 2748.9 [926.1–10159.7], p < 0.001). IgG_SP_ levels increased significantly after vaccine doses 3 and 4 (979.2 [437.6–2250.2], dose 2 vs 9817.75 [4223.1–20352.6] dose 3, p< 0.001; and dose 3 vs 25160.8 [16722.1–40000] dose 4, p<0.001). An increased IgG_NC_ index was also observed after vaccine dose 3 and 4 (0.06[0.03–0.20] dose 2 vs 0.09 [0.05–0.23] dose 3, p <0.001 and dose 3 vs 0.13 [0.06–0.29] dose 4, p = 0.006). Time since the last vaccine received showed a statistically significant effect on IgG_SP_ level. The level of IgG_SP_ in donors who had received the last dose of the vaccine for >90 days was less than those who were vaccinated during this period (1377.8 [521.7–4195.3] vs 11154.7 [3100.1–23371.8] p < 0.001). Interestingly, both IgG_NC_ and IgG_SP_ were higher in donors who were vaccinated with heterologous vaccines (IgG_NC_ index, homologous 0.06 [0.03–0.18] vs heterologous 0.10 [0.05–0.24], p<0.001; and IgG_SP_, homologous 849.0 [379.6–1962.0] vs heterologous 9284.4 [3428.4–28646.0], p<0.001).

**Table 3:** The COVID-19 vaccination regimen and distribution in relation to IgG_SP_.

Donors’ characters	Overall N = 1,520	High IgG_SP_ >1042.6 AU/ml N = 1,140	Low IgG_SP_ <1042.6 AU/ml N = 380	p-value
Vaccination, n (%)	1500 (98.7)	1139 (99.9)	361 (95.0)	<0.001
Vaccine doses, median [IQR]	3 [2–3]	3 [2–3]	2 [2–2]	<0.001
Duration since last dose in days, median [IQR]	75 [35–111]	62 [30–102]	110 [85–133]	<0.001
Duration > 90 days since last dose, n (%)	594	342 (30.0))	252 (66.3)	<0.001
Vaccine dosage, n (%)				
No vaccine	20 (1.3)	1 (0.1)	19 (5.0)	<0.001
1 dose	20 (1.3)	15 (1.3)	5 (1.3)	1.000
2 doses	634 (41.7)	302 (26.5)	332 (87.4)	<0.001
3 doses	698 (45.9)	676 (59.3)	22 (5.8)	<0.001
4 doses	148 (9.7)	146 (12.8)	2 (0.5)	<0.001
Vaccine regimens, n (%)				
SV+SV	20 (1.4)	12 (1.1)	8 (2.3)	0.124
SP+SP	51 (3.6)	6 (0.6)	45 (13.1)	<0.001
AZ+AZ	417 (29.8)	183 (17.4)	234 (68.0)	<0.001
PZ+PZ	9 (0.6)	7 (0.7)	2 (0.6)	1.000
MD+MD	13 (0.9)	13 (1.2)	0 (0)	0.037
AZ+PZ	28 (2.0)	22 (2.1)	6 (1.7)	0.002
SV+AZ	84 (6.0)	54 (5.1)	30 (8.7)	0.009
SV+SV+PZ	81 (5.8)	81 (7.7)	0 (0)	<0.001
SV+SV+AZ	238 (17.0)	221 (21.0)	17 (4.9)	<0.001
SV+AZ+PZ	27 (1.9)	27 (2.6)	0 (0)	0.005
SV+AZ+MD	10 (0.7)	10 (0.9)	0 (0)	0.067
SV+AZ+AZ	15 (1.1)	15 (1.4)	0 (0)	0.030
SP+SP+AZ	14 (1.0)	13 (1.2)	1 (0.3)	0.121
SP+SP+PZ	24 (1.7)	24(2.3)	0(0)	<0.001
SP+SP+MD	17 (1.2)	17 (1.6)	0 (0)	<0.001
AZ+AZ+PZ	178 (12.7))	177 (16.8)	1 (0.3)	<0.001
AZ+AZ+MD	59 (4.2)	59 (5.6)	0 (0)	<0.001
SV+SV+AZ+PZ	36 (2.6)	36 (3.4)	0 (0)	<0.001
SV+SV+AZ+MD	22 (1.6)	22 (2.1)	0 (0)	0.011
SV+SV+PZ+PZ	49 (3.5)	49 (4.6)	0 (0)	<0.001
SV+SV+AZ+AZ	6 (0.4)	6 (0.5)	0 (0)	0.157
Heterologous booster	888 (63.5)	833 (79.0)	55 (21.0)	<0.001

It was observed that the participants who received the whole inactivated virus vaccine (Sinopharm (SP) and /or Sinovac (SV)) as 2^nd^ and 3^rd^ dose showed higher IgG_NC_ S/co index than participants who received the non-whole virus vaccine (Moderna (MD) and /or Pfizer (PZ) and/or AstraZeneca (AZ)) (p<0.001).

When low IgG_SP_ IQR was used as a cut-off value, donors who had IgG_SP_ < 1,042.6 AU/ml were classified as the low antibody group (N = 380) and subjects with an IgG_SP_ level > 1,042.6 AU/ml in the high antibody group (N = 1,140), as shown in Table 3. The median duration of the last vaccination dose was longer in the low-antibody group in comparison to high antibody group (110 [85–133] VS 62 [30–102], p<0.001), and a higher proportion of donors with last dose > 90 days were in the low antibody group (70.8% vs 29.2%, p<0.001). Regarding the number of vaccine doses, a higher proportion of donors who had received only two vaccine doses were found in the low antibody group, especially donors who had received two SP (BBIBP-CorV) vaccines and those who had received two AZ vaccines. Donors who had received three or more doses of vaccine were in the high antibody group, especially heterologous combinations of 2 SV + 1 AZ, 2 AZ + 1 PZ, 2 SV + 1 PZ, and 2 AZ + 1 MD. The lists of different vaccine regimens is stated in Table 3, with a maximum number of donors who received heterologous regimens (888/1500, 59.2.8%), followed by two doses of AZ (417/1500, 27.8%). It was also observed that the MD vaccine in any combination of two, three, or four doses generated a high immune response among the study population.

The multivariate analysis of predictive factors associated with the IgG_SP_ antibody level is stated in Table 4. We observed that age > 50 years, homologous vaccine regimen, and > 90 days duration from the last vaccination are independent predictors associated with low IgG_SP_ < 1042.6 AU/ml, while past COVID-19 infection and three or more vaccine doses prevents low IgG_SP_ < 1042.6 AU/ml.

**Table 4:** Factors associated with low IgG_SP_ <1042.6 AU/ml.

Predictors	Univariate analysis	p-value	Multivariate analysis	p-value
	Relative risk (95% CI)		Relative risk (95% CI)	
Age > 50 yrs	1.08 (1.00–1.17)	0.032	1.39 (0.95–2.05)	0.094
Past COVID-19 infection	0.87 (0.79–0.96)	0.026	0.27 (0.14–0.55)	<0.001
Homologous vaccine	2.11 (1.91–2.35)	<0.001	3.24 (2.15–4.87)	<0.001
Vaccine ≥ 3 doses	0.49 (0.45–0.53)	<0.001	0.08 (0.04–0.13)	<0.001
Duration since last vaccination > 90 days	1.53 (1.42–1.65)	<0.001	4.12 (2.99–5.69)	<0.001

## Discussion

In this cohort of 1,520 healthy blood donors, the seroprevalence study of SARS-CoV-2 antibodies from December 2021 to March 2022 revealed multiple significant key insights regarding donors’ immunity either from immunization or infection. Most of the donors had vaccinations, and nearly all of them (97.6%) tested positive for the spike antibody (IgG_SP_). However, the antibody level varied, and the significant factors that affected these levels were past SARS-CoV-2 infection, heterologous booster vaccines, vaccine ≥ 3 doses, and duration since the last vaccination > 90 days. A total of 84 donors had a history of COVID-19 infection. Of these, 46 (54.8%) had detectable IgG_NC_ and all of these donors were vaccinated.

Ebinger JE et al. [19] had reported that past SARS-CoV-2 infection is one of the dominant factor affecting IgG_SP_ levels in their study. They observed that immune response of individuals with past infection history after only one mRNA vaccination, was similar to individual with no past infection history and with two doses of vaccine. In our study, out of 84 participants with past infection, only 4 participants had received one dose of vaccine, rest all had received 2 or more doses of vaccine. When compared, IgG_SP_ levels between the past history of infection cohort and with no history of infection, the median IgG_SP_ of past infections was higher but was not statiscally significant. Using univariate and multivariate analysis, our results indicated that past COVID-19 infection prevents low IgG_SP_ < 1042.6 AU/ml. Similar to Ebinger JE et al. [19], we also observed that the past infection history cohort with only one or two doses of vaccine could achieved higher IgG_SP_ response. However, recipients of three or more doses of vaccine with the past infection history did not show any additional effect (S1 Table).

The impact of using different types of COVID-19 vaccines on antibody levels was remarkable in the study cohort, and there are increasing numbers of such reports for the same. Various vaccine types used in Thailand, as an initial vaccination, consisting of inactivated virus vaccines (Sinovac, and Sinopharm), viral vector vaccines (ChAdOx1) and mRNA vaccines (Moderna’s mRNA-1273 and Pfizer–BioNTech’s Comirnaty BNT162b2) since the beginning of the COVID-19 pandemic, while boosters were mainly viral vector and mRNA types. A previous study performed in a limited number of healthy volunteers from our institution reported that the use of heterologous vaccination was beneficial and BNT162b2 as a boosting agent after CoronaVac or ChAdOx1 was very immunogenic [20]. A study from the US was conducted on adults who had completed a Covid-19 vaccine regimen for at least 12 weeks [13]. Subjects were assigned to receive the original vaccine and a booster injection with one of three vaccines: BNT162b2(Pfizer), mRNA-1273 (Moderna), Ad26.COV2.S (Johnson & Johnson–Janssen). They reported that both homologous and heterologous boosters resulted in increased neutralizing antibody titers and spike-specific T-cell response. The findings of our study also confirm a good immune response from the use of heterologous vaccines. However, there was no data available to compare third and fourth doses of mRNA vaccines. From our study results, it can be concluded that heterologous vaccines may be considered when mRNA vaccine was not used as primary vaccine.

Another important finding in our study was the immune response after the number of vaccine doses received. Vaccinees with 3 or more doses showed higher levels of IgG_SP_ than the others. This finding supports prior reported clinical findings. Yoon et al. [21]. Published a short report on protection against SARS-CoV-2 with the third dose of mRNA vaccine. They observed that the third dose of vaccine provided strong protection against the delta variant (91%) and moderate protection against omicron infection (64%). A placebo-controlled, randomized trial which assigned participants who received two 30-μg doses of the BNT162b2 vaccine at least six months earlier to be injected with a third dose of the BNT162b2 vaccine or with placebo [22] observed much lower COVID-19 infection in participants in the vaccine group than in the placebo group, which corresponded to a relative vaccine efficacy of 95.3% (95% confidence interval, 89.5 to 98.3). Similarly, in a cohort study of 10.6 million residents in North Carolina [23], the receipt of boosters was associated with a lower risk of SARS-CoV-2 infection (including Omicron) and the resulting hospitalization and death. Our findings add laboratory evidence to such and many more clinical findings reported until now.

Finally, we found that the duration longer than 90 days after the last vaccine affected the level of spike antibodies, which was also reported in other studies. In a longitudinal prospective study involving vaccinated health workers [24] who received two doses of vaccine, monthly tests for the level of anti-spike IgG and neutralizing antibodies were performed. Within six months, the IgG_SP_ antibodies decreased at a consistent rate, whereas the level of neutralizing antibodies decreased rapidly for the first three months with a relatively slow decrease thereafter. A case-control study from Qatar [25] estimated that the effectiveness of BNT162b2 against any SARS-CoV-2 infection was negligible in the first two weeks after the first dose and reached its peak in the first month after the second dose. Effectiveness decreased gradually thereafter, with the decline accelerating after the fourth month to reach approximately 20% in months 5 through 7 after the second dose.

As SARS-CoV-2 vaccines continue to be rolled out globally, individuals and their physicians may wish to seek reassurance that vaccination is ’effective’ and understand how long protection is likely to last [26]. Given that current vaccines generate an immune response to viral spike antigens, anti-spike antibody titers, associated with neutralizing activity, provide a potential surrogate marker of protection [27]. Therefore, understanding the assay- and time-dependent dynamics of post vaccine anti-spike antibodies, understanding differences between individuals with different age, gender and comorbidities, and how these findings relate to protection, is increasingly important.

For IgG_NC_, which should identify natural SARS-CoV infection, it was positive in only 46 of 84 (56%) with a known history of SARS-CoV-2 infected cases in this study. This may be due to the possibility of mild cases of COVID-19 infection in which up to 22% could have negative IgG_NC_ and/or the waning of IgG_NC_ from the long interval between COVID-19 infection and the date tested, as reported by Van Elslande et al. [28]. They observed that the positive IgG_NC_ went down from 85% at four months to 70% at six months and 30% at one-year post infection. In our study, forty percent of previously infected participant had infections more than 6 months prior to the sample collection, which explains the negative IgG_NC_ percentage in the infection history cohort. However, the IgG_NC_ helped detect additional probable cases of asymptomatic infection in the participant who reported no history of SARS-CoV-2 infection in our study. Added together, the number of past COVID-19 infections in our study should be at least 116 cases (7.63%)–calculated from 84 positive history and 36 IgG_NC_ positive cases without a history of infection. Hence, 31% of the probable infections assumed by IgG_NC_ results in our blood donors were asymptomatic infection.

Although we observed differences in the proportion of the magnitude of the immune response following different vaccination regimens, this should not be taken alone as evidence that one vaccine is likely to be more effective than another. Anti-spike antibody titers are associated with neutralizing activity, but the degree to which quantitative anti-spike results are a surrogate for protection against infection, or other endpoints of interest such as hospitalization, death, or onward transmission, remains unclear.

The limitations of the study included no information about the severity of previous SARS-CoV-2 infection and the use of a single assay to quantify post-vaccine anti-spike antibody levels. However, as it is commercially available and well-calibrated, results should be generalizable. Furthermore, we focused on blood donors, who were supposed to be a healthy population. As a result, the ability to assess variations in children or those whose age >65 years was limited.

## Conclusions

We reported immunological responses to COVID-19 vaccines and natural infection in blood donors. Anti-spike antibodies (IgG_SP_) response to vaccines was determined. Markedly higher immune response to vaccines was observed after SARS-CoV-2 infection. At least one booster dose after two initial vaccinations was important to ensure a high antibody level, and the use of heterologous vaccination was beneficial. Finally, IgG_SP_ levels waned after 90 days, indicating that additional vaccination may be needed in the future. Quantitative IgG_SP_ is useful to evaluate antibody response from both vaccination and previous SAR-CoV-2 infection and to identify individuals who need further action, such as booster vaccination dose or change of vaccine regimen.

## Supporting information

S1 Table IgG_SP_ level wrt vaccine doses & past SARS-CoV-2 infection history. (DOCX)

S1 File Supporting data file for “Anti-SARS-CoV-2 antibody among SARS-CoV-2 vaccinated vs post-infected blood donors in a tertiary hospital, Bangkok, Thailand”. (XLSX)
