# Supplementary material for: Anti-SARS-CoV-2 antibody among SARS-CoV-2 vaccinated vs post-infected blood donors in a tertiary hospital, Bangkok, Thailand
Source: PLoS One. 2023 May 18;18(5):e0285737. doi: 10.1371/journal.pone.0285737 (PMC10194992; doi:10.1371/journal.pone.0285737)
Supplement: S1 Table — (DOCX) [file pone.0285737.s001.docx]

**S1Table. IgG_SP_ Level with related to vaccine doses & prior SARS-CoV-2 infection history**

| **Vaccination** | **Prior Infection** | **Naïve** |
| --- | --- | --- |
| One dose, n  Median IgGSP (IQR) | 7  7,816 ( 3,896-13,489) | 13  1,245(134-2,749) |
| Two doses  Median IgGSP (IQR) | 50  5,267 (1,698-9,398) | 584  897 (399-1,928) |
| Three doses , n  Median IgGSP (IQR) | 22  10,186 (2,984-22,127) | 676  9,628 (4,262-20,203) |
| Four doses , n  Median IgG_SP_(IQR) | 5  >40,001(20,337- >40,000) | 140  25,060 (16,661 – 38,958) |
